# Internal enhancement of DNA damage by a novel bispecific antibody‐drug conjugate‐like therapeutics via blockage of mTOR and PD‐L1 signal pathways in pancreatic cancer

**DOI:** 10.1002/cam4.1974

**Published:** 2019-01-25

**Authors:** Rui Cao, Wenping Song, Cheng Ye, Xiujun Liu, Liang Li, Yi Li, Hongjuan Yao, Xiaofei Zhou, Liang Li, Rongguang Shao

**Affiliations:** ^1^ Key Laboratory of Antibiotic Bioengineering of National Health and Family Planning Commission (NHFPC), Institute of Medicinal Biotechnology (IMB) Chinese Academy of Medical Sciences and Peking Union Medical College (CAMS & PUMC) Beijing China

**Keywords:** ADC‐like therapeutic agent, bispecificity, EGFR, HER2, pancreatic ductal adenocarcinoma

## Abstract

Pancreatic ductal adenocarcinoma (PDAC) is a refractory malignant tumor with poor prognosis, limited chemotherapeutic efficacy, and only about 5% of 5‐year survival rate. We generated a dual‐targeting ligand‐based lidamycin (DTLL) to investigate its efficacy against pancreatic cancer after preparing its precursor, DTLP. DTLP was shown specifically binding to EGFR and HER2 on cell surface, followed by endocytosis into cytoplasm of pancreatic cancer cells. DTLL significantly promoted apoptosis and cell cycle arrest at G2/M stages and inhibited cell proliferation. Pancreatic tumors of either MIA‐paca‐2 cell line‐derived (CDX) or patient‐derived xenograft (PDX) mouse models were significantly regressed in response to DTLL. It suggested that DTLL might be a highly potent bispecific antibody‐drug conjugate (ADC)‐like agent for pancreatic cancer therapy. LDM is known to function as an antitumor cytotoxic agent by its induction of DNA damage in cancer cells, therefore, DTLL, as its derivative, also showed similar cytotoxicity. However, we found that DTLL might reverse the AKT/mTOR feedback activation induced by LDM at the first time. The results from both in vitro and in vivo experiments suggested that DTLL enhanced DNA damage via EGFR/HER2‐dependent blockage of PI3K/AKT/mTOR and PD‐L1 signaling pathways in cancer cells, leading to the inhibition of cell proliferation and immunosurveillance escape from pancreatic tumor. Our studies on DTLL functional characterization revealed its novel mechanisms on internal enhancement of DNA damage and implied that DTLL might provide a promising targeted therapeutic strategy for pancreatic cancer.

## 1 INTRODUCTION

Pancreatic ductal adenocarcinoma is one of the most refractory and rapidly fatal malignant disease with a 5‐year survival of around 4%‐6%.^1^, ^2^, ^3^ Current chemotherapy regimens exhibit only modest survival benefits due to high resistance and the profound symptomatic effects of PDAC.^4^, ^5^ Recently, immunotherapy, including ADCs and fusion proteins, is becoming the current craze for the research focus.^6^, ^7^ Therefore, antibody‐based or ligand‐based molecularly targeted drugs might lead to a breakthrough in the treatment of pancreatic cancer.

Lidamycin (LDM) had been demonstrated as a highly potent antineoplastic antibiotics from streptomyces globisporus（CGMCC No.0704）. It is composed of an active enediyne chromophore (AE, 843 Da) responsible for its highly potent cytotoxicity and a noncovalently bound apoprotein LDP (10 500 Da) that forms a hydrophobic pocket for protecting the chromophore. In addition, AE and LDP can be dissociated and reconstituted in vitro.^8^ According to the structural characteristics of LDM, our institute had previously constructed a series of LDM‐derived fusion proteins consisting of AE and various antibody fragments or oligopeptides targeting specific antigens/receptors in tumors by gene recombination and molecular reconstitution. Among these fusion proteins, Ec‐LDP‐Hr‐AE was generated specifically against EGFR and HER2,^9^ showing potent effectiveness in a variety of cancer cells and xenograft tumors of ovary and esophagus.^10^ In pancreatic cancer, EGFR is overexpressed and continually activated, suggesting that this tumor might be responsive to EGFR‐targeted therapies.^11^, ^12^ Several studies have demonstrated that therapeutic agents simultaneously targeting EGFR and HER2 are promising strategies for pancreatic cancer.^13^, ^14^, ^15^, ^16^

**Figure 1:**
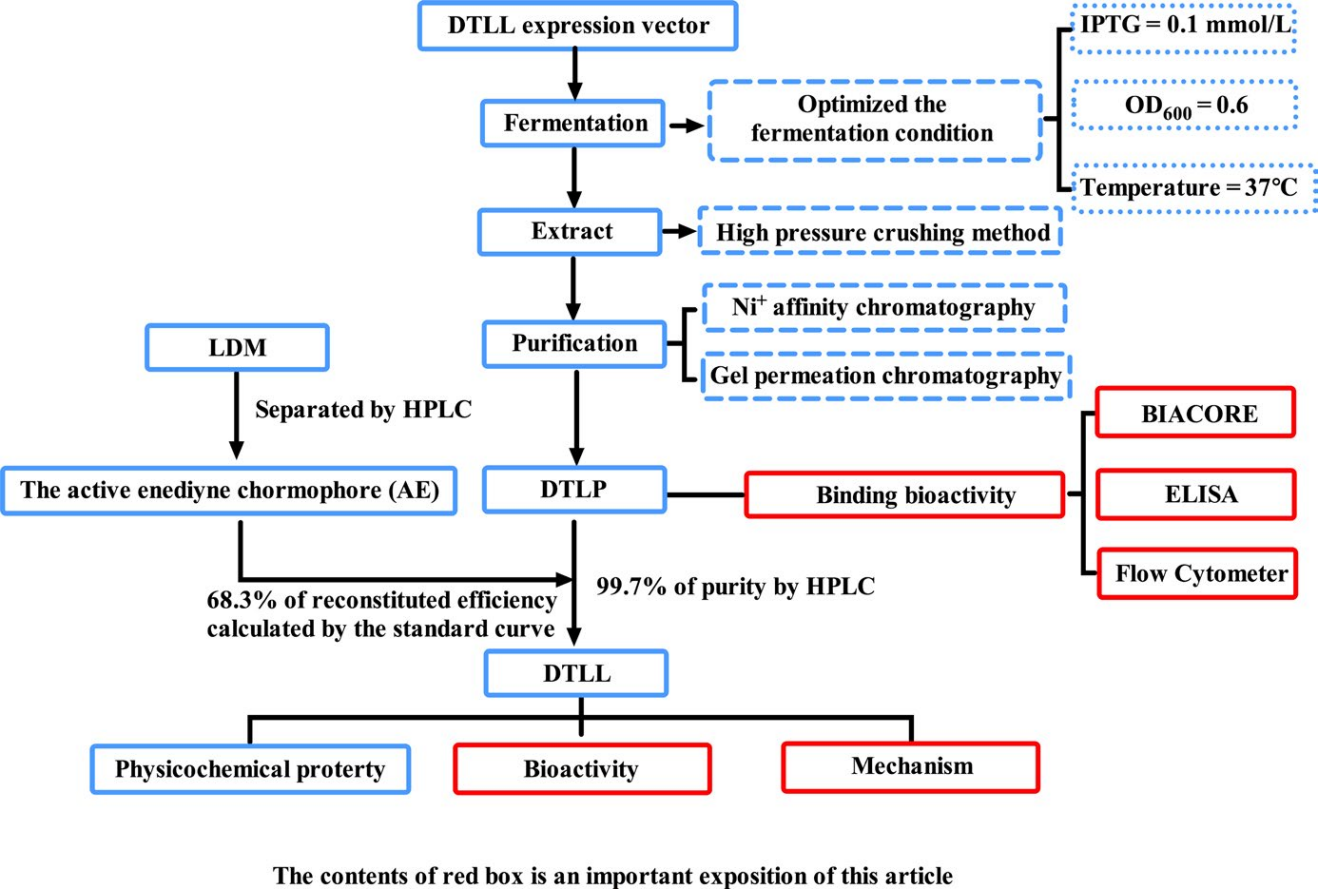
Flowchart for the preparation of DTLP and DTLL

In the present study, we optimized and improved the production methodology of Ec‐LDP‐Hr‐AE to generate this LDM‐derived fusion protein (Shown in Figure 1) renamed DTLL (dual‐targeting ligand‐based LDM) for further investigation in pancreatic cancer, as well as its precursor for DTLP (dual‐targeting ligand‐based LDP). We combined bispecific ligand‐based delivery targeting EGFR/HER2 with the highly effective cytotoxicity of LDM, leading to maximally antineoplastic efficacy of DTLL with reduced side effects. This approach is also in line with the current therapeutic strategy of combination therapy in cancer.

## 2 MATERIALS AND METHODS

### 2.1 Preparation of DTLP and DTLL

We prepared DTLP according to the previous approach (Patent Publication No. CN101497666A) as shown in Figure 1.

### 2.2 Cell lines and antibodies

Human pancreatic carcinoma cell lines AsPC‐1, MIA‐paca‐2, CFPAC‐1, Panc0403, HuP‐T3, and SU86.86 were obtained from Dr Liewei Wang (Department of Molecular Pharmacology of Experimental Therapeutics, Mayo clinic). Cells were cultured in either DMEM (MIA‐paca‐2, CFPAC‐1, and Panc0403) or RPMI 1640 (AsPC‐1, HuP‐T3, and SU86.86) supplemented with 10% fetal bovine serum (FBS; Gibco; Life Technologies, Grand Island, NY, USA), penicillin G (100 U/mL), and streptomycin (100 μg/mL) in an incubator maintained at 37°C with 5% CO_2_.

### 2.3 Biacore assay

Biomolecular analysis of the interaction of DTLP with EGFR/HER‐2 was assessed using the Biacore 2000 biosensor instrument (GE Healthcare, Kontaktuppgifter, Sweden). Immobilization of recombinant EGFR or HER2 (Life Technologies) on a sensor chip CM5 (GE Healthcare) was performed using an amine coupling kit according to the manufacturer's manuals. DTLP solution was injected at doses of 0.29, 0.57, 1.15, 2.29, and 4.58 μmol/L. The resonance angle was measured in resonance units (RU).

### 2.4 Enzyme‐linked immunosorbent assay (ELISA)

ELISA assay was performed to measure the binding efficiency of proteins tested to human pancreatic carcinoma cells, according to the manufacturer's protocol (Tiangen, Beijing, China).

### 2.5 Internalization assay

After incubation with a FITC‐labeled protein at 4°C for 1 hour, cells were rinsed with cold PBS and resuspended to be divided into two samples that were kept at 4 and 37°C, respectively. Cell samples were collected at different times, and 0.04% trypan blue dye was added to quench the extracellular fluorescence for 10 minutes, followed by FACS Calibur flow cytometry (BD Biosciences, San Diego, CA, USA) at 488 nm observed under a fluorescence microscope (Olympus, Tokyo, Japan).

### 2.6 In vivo optical imaging system

After cancer cells were inoculated in the right armpit of athymic mice, FITC‐labeled DTLP was injected into the tail vein. Xenograft mice were subjected to optical imaging at various time points after injection followed by measurement with Living Image software (Xenogen, Astin, TX, USA). After the animals were euthanized, specimens were taken from the tumors and various organs for ex vivo fluorescence imaging observation.

### 2.7 Cell viability assay

Cells seeded in 96‐well plates were treated with different concentrations of tested agents for 48 hours at 37°C, and cell viability was measured with MTS assay according to the manufacturer's manuals. Absorbance was measured at 490 nm using a microplate reader (Thermo Fisher Scientific, Bremen, Germany). For clonogenic assay, tested agents were added into 6‐well plates, and the drug‐containing medium was discarded after 24 hours, followed by an additional 7‐10 days of culture. Cells were fixed with methanol for 15 minutes and stained with crystal violet for clone counting.

### 2.8 Animal study

We used both pancreatic cancer cell line‐derived xenograft (CDX) and patient‐derived xenograft (PDX) mouse models for in vivo evaluation of DTLL efficacy. PDX models were obtained from the HuPrime^®^ allograft PDX platform of the Crown Bioscience Inc (Beijing, China) in which there were RNA sequencing datasets available for each selected mouse model. Four‐ to 5‐week‐old female BALB/c nude mice (Huafukang Bioscience co, Beijing, China) were inoculated with either MIA‐paca‐2 cells (1 × 10^7^/mL) or human pancreatic tumor tissue samples subcutaneously. Animal protocols were approved by the Institutional Animal Care and Use Committee of the Institute of Medicinal Biotechnology (IMB), Chinese Academy of Medical Sciences & Peking Union Medical College (CAMS & PUMC).

### 2.9 TUNEL assay

Sections were incubated with TdT + dUTP at 37°C and counterstained with DAPI (Beyotime Biotechnology, Shanghai, China). The sections were then observed under a microscope (Olympus).

### 2.10 Statistics analysis

Results are expressed as the mean ± SD. Statistical significance was analyzed using ANOVA followed by Student's *t* test (*P* < 0.05).

## 3 RESULTS

### 3.1 DTLP showed bispecific binding activities to EGFR/HER‐2 receptors in pancreatic carcinoma cells

**Figure 2:**
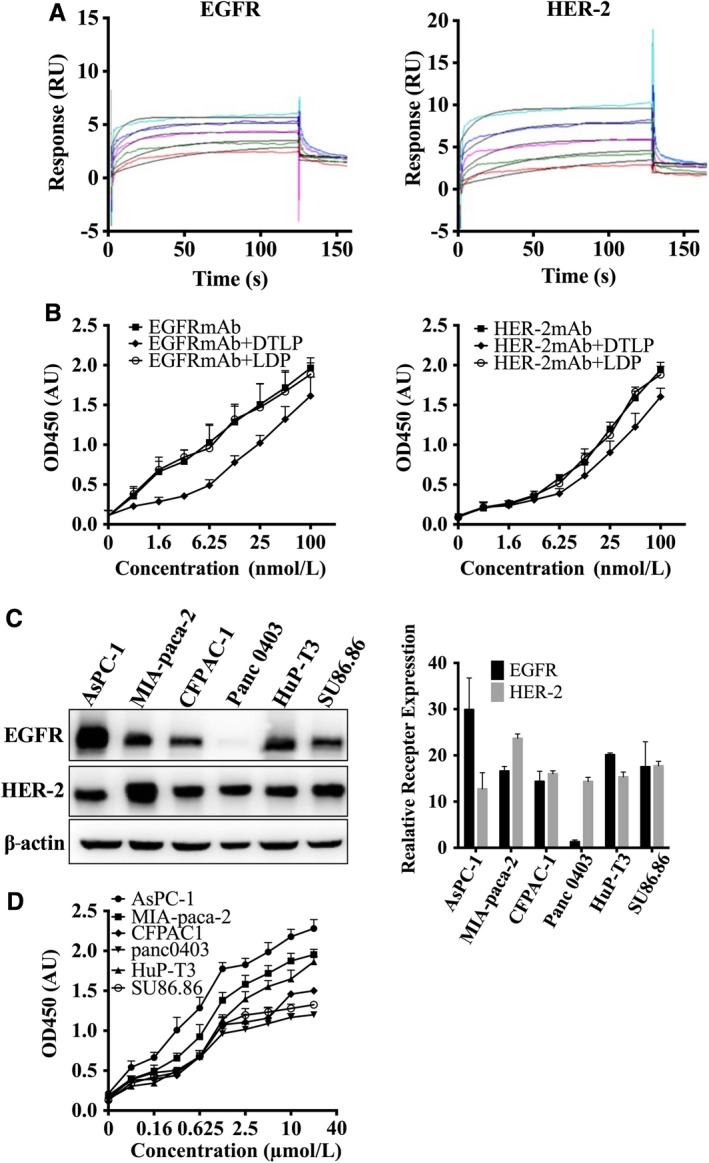
DTLP showed bispecific binding activities to EGFR/HER‐2 receptors in pancreatic carcinoma cells. A, Biacore was used to determine the affinity of the fusion protein DTLP for EGFR and HER2. After coupling EGFR (HER‐2) to a C5 chip, DTLP solution was injected at doses of 0.29, 0.57, 1.15, 2.29, and 4.58 μmol/L at 30 μL/min for 180 s. The *x*‐axis indicates time, and the *y*‐axis represents resonance units (RU). B, Competitive ELISA was used to analyze the competitive affinity of DTLP with EGFR mAb (Left) and HER‐2 mAb (Right). After coating with the receptor, different concentrations of rabbit anti‐EGFR/or anti‐HER‐2 antibody or mixture of antibody and protein were added to incubate for 2 h at 37°C before detection. HRP‐conjugated goat anti‐rabbit IgG were used as the secondary antibody, followed by incubation with TMB used as a substrate solution. C, Protein levels of EGFR and HER‐2 in different pancreatic cell lines analyzed by Western blotting assay, and quantitative evaluation for each line were performed by using GraphPad Prism 6.0 software (GraphPad Software, San Diego, CA, USA). D, The binding affinity of DTLP proteins with different pancreatic cells analyzed by ELISA. Cells were incubated with 50 μL of protein at 4°C overnight. Rabbit anti‐His‐tag antibody and HRP‐conjugated goat anti‐rabbit IgG were used as the primary and secondary antibodies, respectively. For all graphs, error bars indicate SD for n = 3

We first performed a direct binding assay using a Biacore2000 instrument to test whether DTLP binds directly to both EGFR and HER‐2 receptors.^17^ The result confirmed that DTLP apparently bound to both recombinant EGFR and HER‐2 on chips with a series of diluted concentrations (Figure 2A). The kinetic constants (K_D_ values) for binding between DTLP and EGFR/HER2 were 6.84 × 10^−8^ and 7.90 × 10^−8^ mol/L, respectively. Subsequently, competitive ELISA demonstrated that DTLP significantly impaired the binding capabilities between EGFR/HER‐2 and their respective antibodies (Figure 2B). In contrast, no remarkable binding change was observed by adding LDP, confirming the bispecificity of DTLP (Figure S1). Next, we used a variety of pancreatic carcinoma cell lines to perform Western blotting and ELISA assays and select a suitable cell line that best reflects the dual‐targeting function of DTLP. Among these cell lines, AsPC‐1 and MIA‐paca‐2 cells expressed higher levels of EGFR/HER2, while DTLP also showed greater binding activity to those two lines in the ELISA assay. The results indicated that DTLP was able to bind to all those six lines, and its binding affinity was positively correlated with the expression levels of these two receptors in the cells (Figure 2C,D). MIA‐paca‐2 cell line was eventually chosen for follow‐up studies, not only because of its high binding affinity and exquisite specificity to DTLP, but also due to having the most significant cellular sensitivity to DTLL, as determined in MTS assay (Figure S2).

### 3.2 DTLP specifically targeted EGFR/HER2 via its effective internalization into MIA‐paca‐2 cells

**Figure 3:**
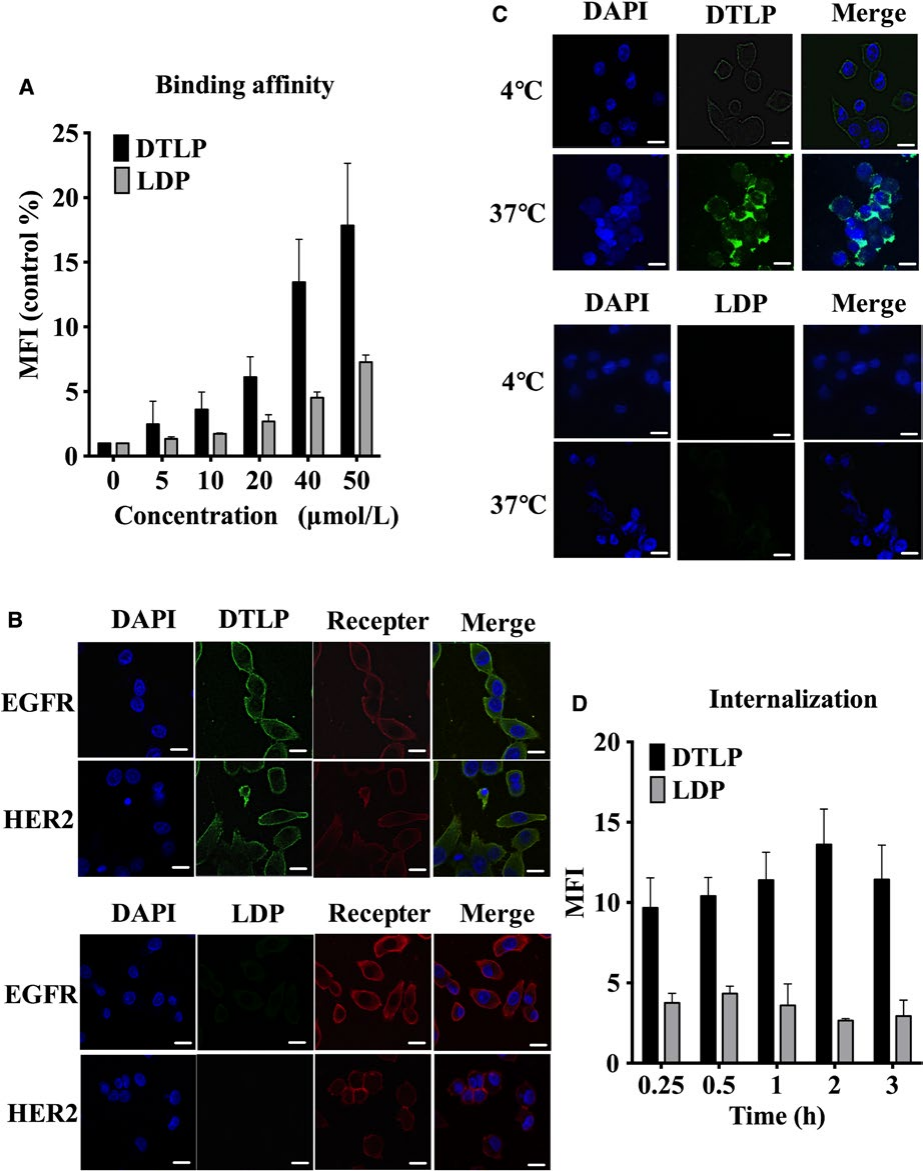
DTLP effectively internalized into MIA‐paca‐2 cells after bispecific binding to EGFR and HER‐2. A, MIA‐paca‐2 cells were incubated with FITC‐labeled DTLP protein and LDP protein at indicated concentrations, and the mean fluorescence intensities (MFIs) were analyzed by flow cytometry. Following FACS, MFI/Control were plotted vs protein concentrations representing three repeated times analyzed with GraphPad Prism 6.0 software. B, Colocalization of DTLP with EGFR (Right) and HER‐2 (Left) on MIA‐paca‐2 cells analyzed by laser scanning confocal microscopy (400×). Immobilized cells were incubated with anti‐EGFR or HER‐2 antibody (1:500 in dilution) as well as 1 μmol/L of FITC‐labeled DTLP/or LDP at 4℃ for 1 h, respectively, followed with fluorescently labeled secondary antibody (1:200 in dilution) added in order. DAPI is shown in blue for nuclei with DTLP/or LDP in green and EGFR and HER‐2 in red, followed by merged images (MERGE) obtained by superimposing DAPI, anti‐EGFR/or HER2 mAbs, and DTLP/LDP. C, Internalization of DTLP in MIA‐paca‐2 cells was detected by laser scanning confocal microscopy (400×). Living cells with 10 μmol/L of FITC‐labeled DTLP/or LDP were incubated for 1 h at 4 and 37°C successively to allow the proteins into cells. Blue indicates nuclei stained with DAPI, while green represents DTLP/or LDP labeled with FITC. MERGE indicates superimposed DAPI and DTLP/LDP. D, Internalization of FITC‐labeled DTLP/LDP in MIA‐paca‐2 cells at various times detected by flow cytometry. After incubation with FITC‐labeled proteins at 4°C for 1 h, cells were resuspended at 37°C and collected to measure their fluorescence intensity at various times. Following FACS, MFI/Control was plotted vs times after three repeated experiments. For all graphs, error bars indicate SD for n = 3. SD values were calculated with GraphPad Prism 6.0 software. Scale bars indicate 20 μm

Next, our study indicated that FITC‐labeled DTLP showed stronger binding to MIA‐paca‐2 cells in a concentration‐dependent manner by flow cytometry assay, as compared to the signal with FITC‐labeled LDP tested as a negative control (Figure 3A). DTLP specifically stained on the cellular membrane and colocalized with EGFR/HER2 receptors of MIA‐paca‐2 cell surface in immunofluorescence assays (Figure 3B). However, this phenomenon was not observed when using LDP instead of DTLP. The internalization assay (Figure 3C) showed that DTLP bound to the membrane of MIA‐paca‐2 cells at 4°C and was apparently internalized into the cytoplasm through EGFR/HER2‐mediated endocytosis at 37°C, allowing DTLP to efficiently guide cytotoxic effectors, the active enediyne chromophore of LDM (AE), into cells. Moreover, quantitative analysis in flow cytometry assays showed similar results, with gradually increasing FITC signals of cytoplasmic DTLP in a time‐dependent manner, whereas no signal was observed with LDP as a negative control (Figure 3D).

### 3.3 DTLP is specifically enriched in pancreatic tumors

**Figure 4:**
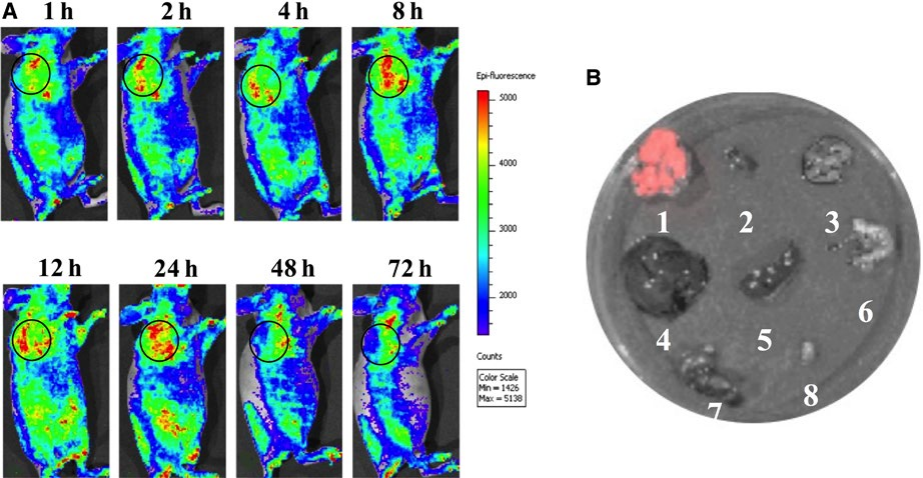
DTLP specifically enriches in tumor tissues of MIA‐paca‐2 CDX mouse models. A, Fluorescent images of tumors in MIA‐paca‐2 xenograft mice were obtained at different times after tail vein injection of FITC‐labeled DTLP at 15 mg/kg. Green fluorescence represents FITC‐labeled DTLP, with black circles indicating tumor areas. Color scale represents photons/s/cm^2^/steradian. After the volume of inoculated tumor reached approximately 300 mm^3^, FITC‐labeled DTLP was injected into the tail vein of mice (n = 3) at dosages of 15 mg/kg. Xenograft mice were subjected to optical imaging at various time points after injection: 1, 2, 4, 8, 12, 24, 48, and 72 h. B, Tissues including tumor, heart, lung, liver, spleen, stomach, kidney, and bladder of mice from (A) were excised and treated, as described in (A) after 72 h of treatment of FITC‐labeled DTLP. Distribution of fluorescent signals was detected with numbers labeling in vitro imaging analysis. The color bar was scored gradually according to the fluorescent intensity

We further observed the distribution of fluorescent signals for FITC‐labeled DTLP in MIA‐paca‐2 CDX mouse models using an optical molecular imaging system to determine whether DLTP specifically targets tumor parenchyma in vivo (Figure 4A). One hour after DTLP intravenous injection, its fluorescent signal started to become enriched within the tumors, reaching its peak at 24 hours. At 72 hours after injection, we further detected DTLP fluorescent signals in tumor and organs, including heart, liver, spleen, stomach, intestine, kidney, and lung in ex vivo imaging tests. As Figure 4B displayed, the signal was mainly found in the tumor, while no detectable signal was observed in the other organs. Therefore, we demonstrated that DTLP was specifically guided into pancreatic tumors by using both in vitro and in vivo models (shown in Figures 3 and 4).

### 3.4 DTLL promoted cell cycle arrest and apoptosis, leading to inhibition of proliferation

**Figure 5:**
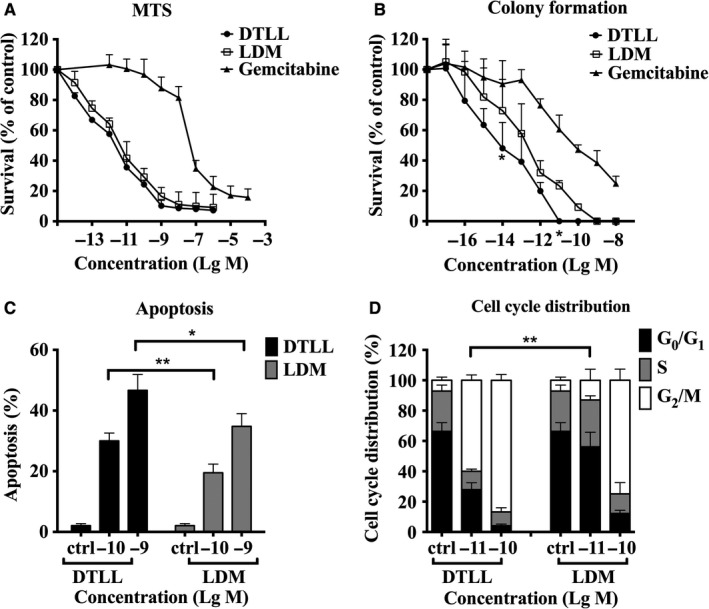
DTLL promotes inhibition of proliferation, cycle arrest and apoptosis of pancreatic cancer cells. A, The inhibition rate of MIA‐paca‐2 cell growth was detected by MTS assay. Cells were treated with different concentrations of tested agents including DTLL, LDM, and gemcitabine at indicated concentrations for 48 h at 37°C. B, The inhibition rate of MIA‐paca‐2 cell growth was detected using a clonogenic assay with the same treatments as (A) except that the medium was replaced with drug‐free medium after 24 h. C, Flow cytometry analysis was performed to measure apoptosis of MIA‐paca‐2 cells induced by DTLL/or LDM at 0.1 and 1 nmol/L for 24 h. D, MIA‐paca‐2 cells were exposed to DTLL and LDM at different concentrations (0.01 and 0.1 nmol/L), and cell cycle distribution was determined by flow cytometry after PI staining. The SD values for three repeated experiments were calculated with GraphPad Prism 6.0 software

After demonstrating the EGFR/HER2‐mediated bispecificity of DTLP in pancreatic cancer cells, we used DTLP and AE (the chromophore of LDM) to produce DTLL and test whether DTLL exhibited potent antineoplastic bioactivity for pancreatic cancer therapy. In our study, DTLL showed stronger cytotoxicity to MIA‐paca‐2 cells in both MTS (Figure 5A) and colony formation (Figure 5B) assays, as compared to that of gemcitabine (*P* < 0.05). Moreover, DTLL cytotoxicity was significantly greater than that of LDM in the colony formation assay (*P* < 0.05) in which those drugs were removed after 24 hours of treatments. DTLL was able to be immediately internalized into tumor cells guided by EGFR/HER2, whereas LDM reached the inside of the cells only after its passive endocytosis, therefore DTLL was able to function more effectively.

Our results found that apoptosis was significantly induced by DTLL and LDM in a dose‐dependent manner. The percentage of apoptotic cells induced by 1 nmol/L of DTLL was greater than that by LDM (46.68% vs 34.8%, respectively), as well as 30.0% vs 19.5% at 0.1 nmol/L (Figure 5C). Subsequently, the distribution of cell cycle was evaluated with PI staining in flow cytometry analysis. MIA‐paca‐2 cells in G_2_/M phase significantly increased with exposure to either DTLL or LDM in a dose‐dependent manner (Figure 5D). DTLL significantly caused more cells (59.96%) to arrest in G2/M phase, even at the lower dose of 0.01 nmol/L, as compared to LDM (13.02%). It suggested that DTLL promoted cell arrest and apoptosis, contributing to better anti‐proliferative effects on MIA‐paca‐2 cells.

### 3.5 DTLL repressed tumor growth in both CDX and PDX mouse models

**Figure 6:**
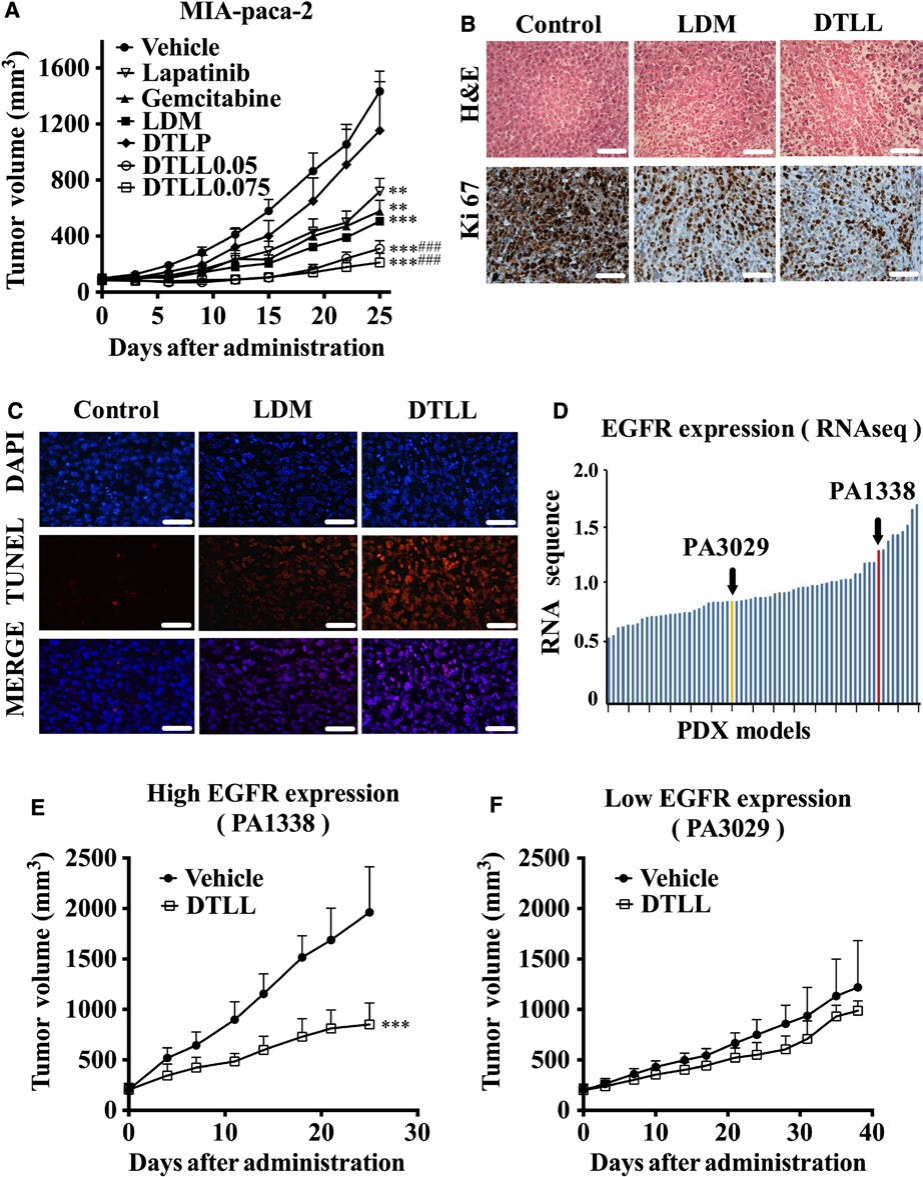
DTLL repressed pancreatic carcinoma growth of CDX and PDX mouse models. A, Nude mice (n = 5) bearing human pancreatic carcinoma MIA‐paca‐2 xenograft were treated with gemcitabine (ip 60 mg/kg), LDM (iv 0.05 mg/kg), DTLP (iv 1 mg/kg), or DTLL (iv 0.05 or 0.075 mg/kg of the LDM‐equivalent dose) on Day 0 and Day 10 after tumor formation, respectively. Lapatinib (ig 75 mg/kg) was given once a day. The mean values of tumor volumes in each group are shown with error bars for SD values (n = 5). **P < 0.01 when compared each group with the control while ***P < 0.001. ^###^ *P* < 0.001 compared with the LDM‐treated group. B, Sections from paraffin‐embedded MIA‐paca‐2 xenograft tumors were stained by Hematoxylin and Eosin (H&E) (upper panels) and Ki‐67 IHC (lower panels) (×400). Scale bars indicate 100 μm. C, Apoptosis in MIA‐paca‐2 xenograft tumors was measured using TUNEL assay. TUNEL positive cells shown in red fluorescence were detected, and DAPI is shown in blue. MERGE represents merged DAPI and TUNEL signals. Scale bars indicate 100 μm. D, RNA sequencing data from tumor samples of HuPrime^®^ PDX models in a variety of pancreatic carcinoma patients were plotted to determine expression levels of EGFR mRNA with two selected models, PA1338 in red and PA3029 in yellow, shown by arrows. For efficacy evaluation, two selected PDX models with high (E) and low (F) levels of EGFR expression were administered in nude mice (n = 5) with vehicle or DTLL at the LDM‐equivalent dose of 0.1 mg/kg once a week for 3 wk, respectively. Tumor volumes were measured after animals were sacrificed on Day 24 and 39, respectively. ***P < 0.001.

Human pancreatic carcinoma MIA‐paca‐2 cells were used to generate an in vivo CDX mouse model and to evaluate antineoplastic efficacy of DTLL, vehicle, LDM, DTLP, gemcitabine, and lapatinib controls. As shown in Figure 6A, Table 1, and Figure S3, DTLL significantly repressed the tumor growth by 85.9% at the LDM‐equivalent dose of 0.05 mg/kg, as well as showing an 88.9% inhibitory rate at 0.075 mg/kg. LDM inhibited tumor growth by 68.1% at dose of 0.05 mg/kg, its maximal tolerated concentration, statistically significantly lower than in the DTLL‐treated group (*P* < 0.001). At the end of the experiment, no toxico‐pathological changes were observed in organs of mice treated with DTLL and LDM (Figure S4A). Subsequently, we observed strong IHC staining of Ki‐67, a marker of cell proliferation, in vehicle‐treated tumors, whereas only weak and moderate staining was observed in DTLL‐ and LDM‐treated tumors, respectively. These data indicated that DTLL had more significant anti‐proliferative activity in vivo (Figure 6B). In addition, we confirmed the anti‐apoptotic effects of DTLL were greater than LDM using TUNEL staining (Figure 6C). Our results suggest that DTLL showed highly potent efficacy against pancreatic carcinoma in in vivo CDX models as compared to LDM. DTLP alone showed little therapeutic benefit, with a 19.7% inhibition rate, even at the dose of 1 mg/kg.

**Table 1:** Therapeutic efficacy of DTLL against pancreatic carcinoma MIA‐paca‐2 CDX mouse models

Treatment groups	Number of mouse	Tumor weight (g) Mean ± SD	Inhibition rate (%) Mean ± SD
Control	5	1.47 ± 0.5	—
Lapatinib	5	0.66 ± 0.19	55.2 ± 12.6^a^
Gemcitabine	5	0.57 ± 0.16	61.2 ± 10.7^a^
LDM	5	0.47 ± 0.08	68.1 ± 5.5^b^
DTLP	5	1.18 ± 0.81	19.7 ± 55.3
DTLL 0.05	5	0.21 ± 0.05	85.9 ± 3.7^b^, ^c^
DTLL 0.075	5	0.16 ± 0.07	88.9 ± 4.8^b^, ^c^

Nude mice (n = 5) bearing MIA‐paca‐2 tumors were treated gemcitabine (ip 60 mg/kg), LDM (iv 0.05 mg/kg), DTLP (iv 1 mg/kg), and DTLL (iv 0.05 or 0.075 mg/kg of the LDM‐equivalent dose) on Day 0 and Day 10 after tumor formation, respectively. Lapatinib (ig po, 75 mg/kg) was given once a day.

a *P* < 0.01, compared with the control.

b *P* < 0.001, compared with the control.

c *P* < 0.001, compared with the LDM group.

PDX models derived from fresh human tumor tissue have been widely used in the fields of oncological and pharmacological therapeutics as an effective study tool for translational medicine.^18^ We hence utilized these models to evaluate DTLL efficacy based on *EGFR* and *HER‐2* expression levels that were obtained from RNA sequencing datasets available. Two PDX models, PA1338 and PA3029, were selected due to their differences in EGFR expression prominently, not on that of HER2 level due to the limited models available (shown in Figure 6D and Figure S5). As observed in the EGFR‐high‐expression model (PA1338), tumors of DTLL‐treated mice reached an average volume of 850.59 mm^3^ after 3 weeks of administration. The inhibitory rate was 56.63%, significantly less than that in the vehicle controls (1961.25 mm^3^; *P* < 0.001) (Figure 6E and Table 2). However, in the EGFR‐low‐expression model (PA3029), the maximum inhibitory rate by DTLL was only up to 29.44% on Day 39, showing a smaller trend in tumor volume than vehicle group (Figure 6F and Table 2). These data indicated that the therapeutic effect of DTLL on the inhibition of tumor growth was closely related to *EGFR* expression levels in tumors. In addition, there were no deaths or significant changes in body weight observed in mice from either treatment group, suggesting its safety even at LDM‐equivalent therapeutic doses of 0.1 mg/kg (Figure S4B).

**Table 2:** Therapeutic efficacy of DTLL against human pancreatic carcinoma PDX models

PDX models	Treatment groups	Number of mouse	Tumor volume (mm^3^) Mean ± SD	Inhibition rate Mean ± SD
PA1338 (High EGFR)	Vehicle	5	1961.25 ± 202.51	—
	DTLL	5	850.59 ± 95.25	56.63 ± 9.71^a^
PA3029 (Low EGFR)	Vehicle	5	857.24 ± 81.95	—
	DTLL	5	604.87 ± 59.23	29.44 ± 13.82

PDX mice were administrated vehicle or DTLL at the LDM‐equivalent dose of 0.1 mg/kg once a week for 3 wk. Tumor volumes were measured after animals were sacrificed on Days 24 and 39, respectively. DTLL was administered via tail vein injection once a week for three weeks.

a Compared with vehicle group, *P* < 0.001.

### 3.6 DTLL had an antineoplastic effect through inhibition of EGFR/HER2‐dependent AKT/mTOR signal pathway and PD‐L1‐medicated tumor escape from immunosurveillance

**Figure 7:**
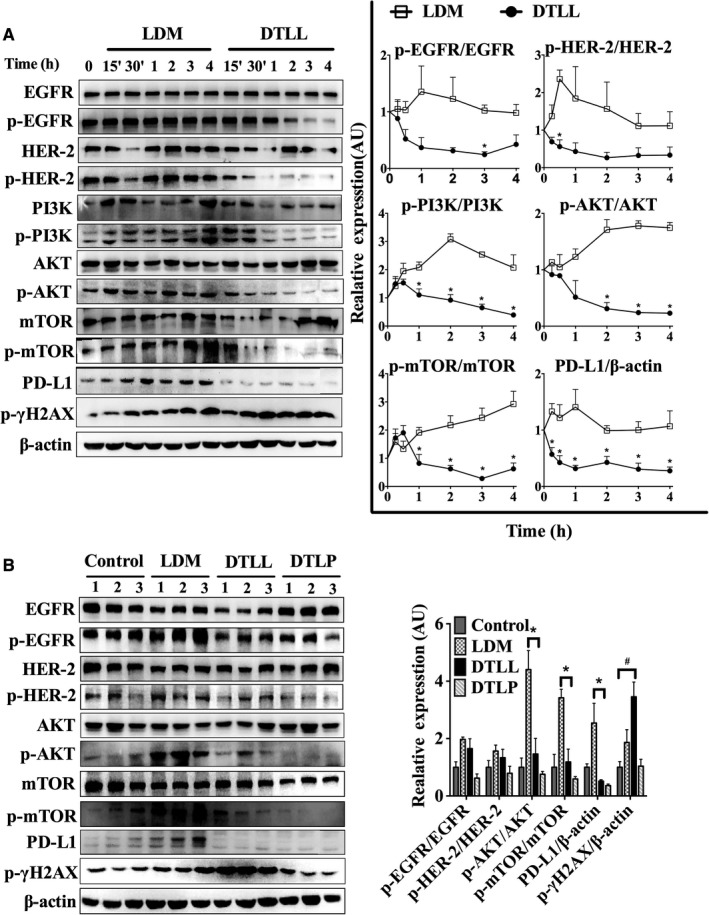
DTLL exhibits an antineoplastic effect through EGFR/HER2‐dependent inhibitions of PI3K/AKT/mTOR signaling and PD‐L1‐mediated escape from immunosurveillance. A, Western blotting assays were used to test protein levels of MIA‐paca‐2 cells treated with DTLL/or LDM at 0.1 nmol/L of the LDM‐equivalent dose for 15 and 30 min, and 1, 2, 3, and 4 h with β‐actin as an internal reference gene. Band intensities were quantified using ImageJ. Data shown are representative of three experiments. Statistical significance was evaluated using unpaired t test using GraphPad Prism 6.0 software. B, Protein levels in tumor samples of MIA‐paca‐2 CDX mouse models were determined by Western blotting. Data were analyzed as described in (A)

After evaluating DTLL therapeutic efficacy, we functionally characterized its mechanism of action in MIA‐paca‐2 cells. Administration of LDM or DTLL for 30 minutes caused DNA damage, showing a significant increase in phosphorylated H2AX, a sensitive target for assessing DNA double‐strand breaks (DSBs) in cells. Our results confirmed that LDM functions as a highly potent cytotoxic agent (Figure 7A). A similar response in phospho‐H2AX was observed in DTLL‐treated samples, indicating one of its molecular mechanisms of action.

When treated with either LDM or DTLL for 15 minutes, the ratios of active phosphorylated and total proteins for HER‐2, EGFR, PI3K, AKT, and mTOR were intermediately increased. However, the following responses in the PI3K/AKT/mTOR signaling pathway to LDM and DTLL were quite different (Figure 7A). Activation of these proteins in LDM‐treated cells remained at higher levels from 30 minutes to 4 hours (Figure 7A). We speculated that the activated PI3K/AKT/mTOR signaling pathways might serve as a responsive feedback to LDM‐induced DSBs in tumor cells. Furthermore, an apparent increase in PD‐L1 expression was detected, suggesting promotion of immune escape by tumor cells to counteract LDM cytotoxic effects. In contrast, there were constitutively inhibitory activations of EGFR and HER2 from 30 minutes to 4 hours in the DTLL‐treated cells, as well as significant decreases in the ratios of p‐PI3K/PI3K, P‐AKT/AKT, and p‐mTOR/mTOR, respectively. Interestingly, we observed a significant decrease in PD‐L1 expression after treatment with DTLL. DTLL's tailored internalization into tumor parenchyma cells after specifically binding to EGFR/HER2 might contribute to blockage of their targeting pathways—the PI3K/AKT/mTOR signaling and PD‐L1‐medicated tumor escape from immunosurveillance. Consequently, we speculated that DTLL might inhibit PI3K/AKT/mTOR signaling activation and PD‐L1‐mediated immune escape in tumors induced by reconstituted AE from DTLL. These data implied potential mechanisms by which DTLL showed more significant antineoplastic efficacy than LDM alone.

Similar results using the tumor samples from the MIA‐paca‐2 CDX model for in vivo evaluation were observed (Figure 6A‐C). There were significant decreases in p‐AKT/AKT, p‐mTOR/mTOR, and PD‐L1 in DTLL‐treated tumor samples with the same inhibitory trends in the ratios of p‐EGFR/EGFR and p‐HER2/HER2. However, those bioactivities were dramatically enhanced with LDM induction (shown in Figure 7B), suggesting that DTLL might promote deactivation of mTOR and PD‐L1 signaling, contributing to the inhibition of cell proliferation and apoptosis in pancreatic carcinoma seen in the in vivo experiments (Figure 6A‐C). Moreover, DTLL induced an increase in phosphorylated ᵧ‐H2AX more than LDM alone, confirming that DTLL inhibited tumor growth much more significantly than LDM.

In addition, little increase in ᵧ‐H2AX was observed in DTLP‐treated tumors, confirming that it functioned as a targeting carrier rather than an antineoplastic agent (Figures 7B and 6). There were some differences in the ratios of p‐EGFR/EGFR, p‐HER2/HER‐2, p‐AKT/AKT, and p‐mTOR/mTOR between DTLP‐ and DTLL‐treated samples, suggesting that DTLP showed more significantly and directly inhibitory activities and that the AE‐associated function of DTLL might be in conflict with its effect (Figure 7B).

## 4 DISCUSSION

To date, many targeted therapies for pancreatic cancer could be accomplished by using specific mAbs, antibody fragments, ADCs, and immuno‐fusion proteins or small‐molecule inhibitors that target diverse pathway‐specific molecules on tumor cell surfaces and within normal tissue stroma.^6^, ^19^ Most were initially designed to inhibit only one or a partial signaling pathway.^20^, ^21^ More preclinical studies have recently focused on strategies to generate bispecific or multivalent targeted agents,^22^, ^23^, ^24^, ^25^ as well as combinations of these agents with chemotherapy.^26^, ^27^ However, many of these therapies are too toxic to be used in targeted therapeutic agents.^28^ Immuno‐fusion proteins are homogeneous and made from a fusion gene by gene recombination.^29^ They act in a similar way to ADCs but avoid some ADC disadvantages, including heterogeneity, narrowed therapeutic windows, and predominantly pharmacokinetic effects.^30^

Based on the findings above, we adopted a dual‐targeted strategy of fusion proteins to successfully prepare DTLL as a bispecific ADC‐like immunotherapeutic agent with reduced side effects by delivering AE directly to pancreatic tumors in the present study. We found that the therapeutic efficacy of DTLL was significantly better than LDM at the dose of 0.05 mg/kg, which is the maximal therapeutic dose for LDM in the CDX models (Figure 6A). Greater benefits were observed after treatment with DTLL even at higher doses (0.075 mg/kg of the LDM‐equivalent dose) without obvious toxicity compared to the LDM alone group (Figure 6A and Figure S4). Results in the clinically relevant PDX models similarly showed that DTLL was more efficacious at 0.1 mg/kg with an acceptable safety profile (Figure 6E and Figure S4). Our study demonstrated that DTLL was superior to free LDM alone in pancreatic cancer in both in vivo and in vitro, similar to observations from previous reports in the treatments of ovarian carcinoma^9^ and esophageal cancer.^10^ In the present study, we selected the PDX model (PA1338) with a higher level of EGFR expression in pancreatic tumor to confirm that DTLL had potent antineoplastic efficacy (Figure 6E), further indicating that its highly potent antitumor activity was predominately attributed to its specifically targeting EGFR.

Our mechanistic studies demonstrated for the first time that DTLL might suppress pancreatic tumor progress by EGFR/HER2‐dependent blockage of AKT/mTOR signaling and PDL1/PD1‐medicated escape from immunosurveillance simultaneously, unlike LDM alone as a cytotoxic agent. This implied that DTLL might reverse the acquisition of drug resistance induced by AE and partially explained why DTLL showed more significant anti‐proliferative activity than LDM alone, which needs for future elucidation. We speculated that DTLL might contribute to inhibition of EGFR‐associated PD‐L1/PD1 signaling, which helped to re‐activate and recruit CD^4+^/CD^8+^ effector cells to the tumor microenvironment for killing tumor cells.^31^, ^32^ Further characterization studies will be required to elucidate its mechanism of action. Certainly, more CDX and PDX models will be needed to precisely distinguish DTLL efficacy in tumors for clinical implementation in the future, according to distinct levels of EGFR and HER2, as well as that of PDL1 and PD1.

The EGFR family (EGFR also known as HER) is a subset of receptor tyrosine kinases that activate and regulate diverse processes, including cell survival, proliferation, differentiation, and migration.^33^, ^34^, ^35^ It has been shown to be activated in many epithelial cancers such as colorectal, breast, and lung cancers.^36^ Overexpression of EGFR has also been identified in pancreatic tumor tissue,^37^, ^38^ especially in recent reports from next‐generation sequencing analysis of tumor tissue samples with pancreatic cancer patients.^39^, ^40^ When combined with clinical data, it suggested that up‐regulation of EGFR was associated with more aggressive tumor behavior, and that its increased activity was also related to higher rates of disease relapse following surgery in pancreatic cancer.^41^ In addition, combination of anti‐EGFR and anti‐HER2 antibodies showed synergistic antitumor efficacy in the treatment of pancreatic cancer.^42^ Therefore, we introduced a bispecific ligand‐based delivery targeting EGFR/HER2 to investigate its effect in pancreatic cancer.

In the current era of precision medicine, overcoming primary and acquired resistance is becoming more and more important to targeted therapy. For instance, small‐molecule tyrosine kinase inhibitors, and monoclonal antibodies targeting certain pathways, suppress the clones that are addicted/dependent on the pathway. However, the initial promising responses are usually followed by disease progression, as other clones which can bypass these signaling pathways through their genetic plasticity might grow rapidly after the initial honeymoon period, eventually leading to progressive disease. The unilateral inhibition of the EGFR family may exist the above drawbacks in the treatment of pancreatic cancer.^43^, ^44^ Partially due to that, many therapeutic agents to block EGFR relevant pathways had little effect on PDAC. In our study, DTLL is designed not only to inhibit activities of both EGFR and HER2, but also to combine with the cytotoxic effect of lidamycin. Therefore, DTLL as a highly potent bispecific antibody‐drug conjugate (ADC)‐like agent might provide a promising targeted therapeutic strategy for pancreatic cancer with little acquired resistance.

There were inevitably some limitations in our study: (a) The fluorescent signals of DTLL distribution from our in vivo targeting imaging test were not very strong or highly specific, being weaker than that of the full‐size antibody probably due to its smaller size and shorter half‐life (*t*_1/2_). (b) DTLL was designed to target EGFR and HER2 within the same ErbB signaling pathway by a similar mechanism of action.^45^ This might make it possible to partially acquire resistance because there would be adaptive responses involved in other potential alternative pathways. Therefore, in follow‐up studies, the combination of dual‐targeted molecules to inhibit different pathways with other chemotherapeutic agents might be more effective. (c) In the present study, although AsPC‐1 cells with higher expression levels of both EGFR and HER2 had the strongest binding affinity to DTLP among the six pancreatic cancer cell lines available (Figure 2D and Figure S1), this line showed intermediate resistance to DTLL and worse efficacy in vitro and in vivo (Data not shown). Therefore, we chose MIA‐paca‐2 with stronger affinity and better response to conduct subsequent experiments. We are currently carrying out genetic analysis of these two lines to identify differential genes involved in drug sensitivity and to characterize potential mechanisms of resistance. (d) We chose available PDX models to evaluate DTLL efficacy mainly due to the prominent difference in EGFR expression, but not HER‐2 expression; therefore, how the expression of HER2 might contribute to DTLL efficacy in pancreatic tumor needs to be further explored using more PDX models with different levels of HER2.^46^

## CONFLICT OF INTEREST

The authors declare no competing interests.
