# Supplementary figures and images for: Internal enhancement of DNA damage by a novel bispecific antibody‐drug conjugate‐like therapeutics via blockage of mTOR and PD‐L1 signal pathways in pancreatic cancer
Source: Cancer Med. 2019 Jan 25;8(2):643–55. doi: 10.1002/cam4.1974 (PMC6382721; doi:10.1002/cam4.1974)

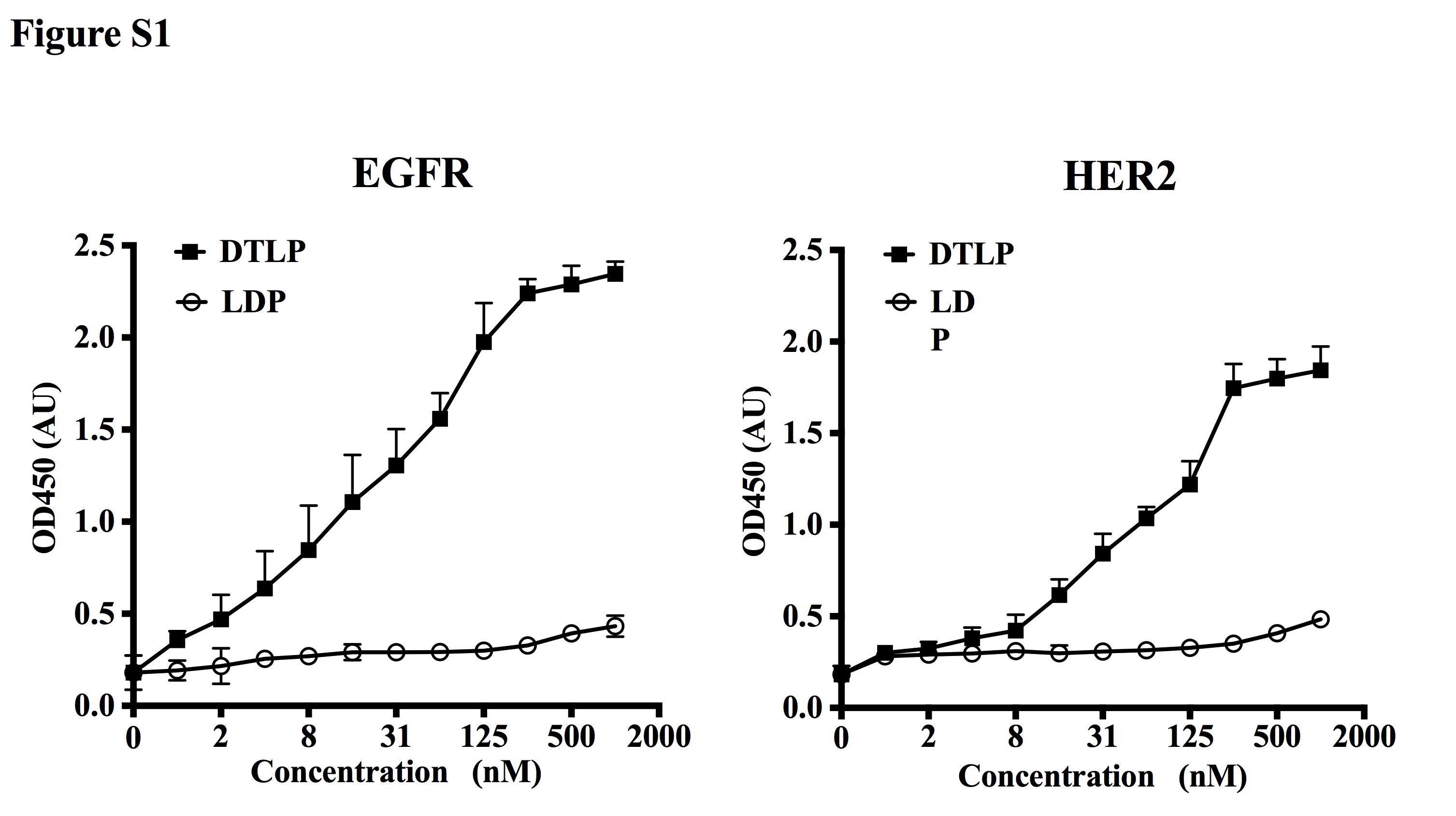

Supplement: Supplementary file 1 [file CAM4-8-643-s001.tiff]

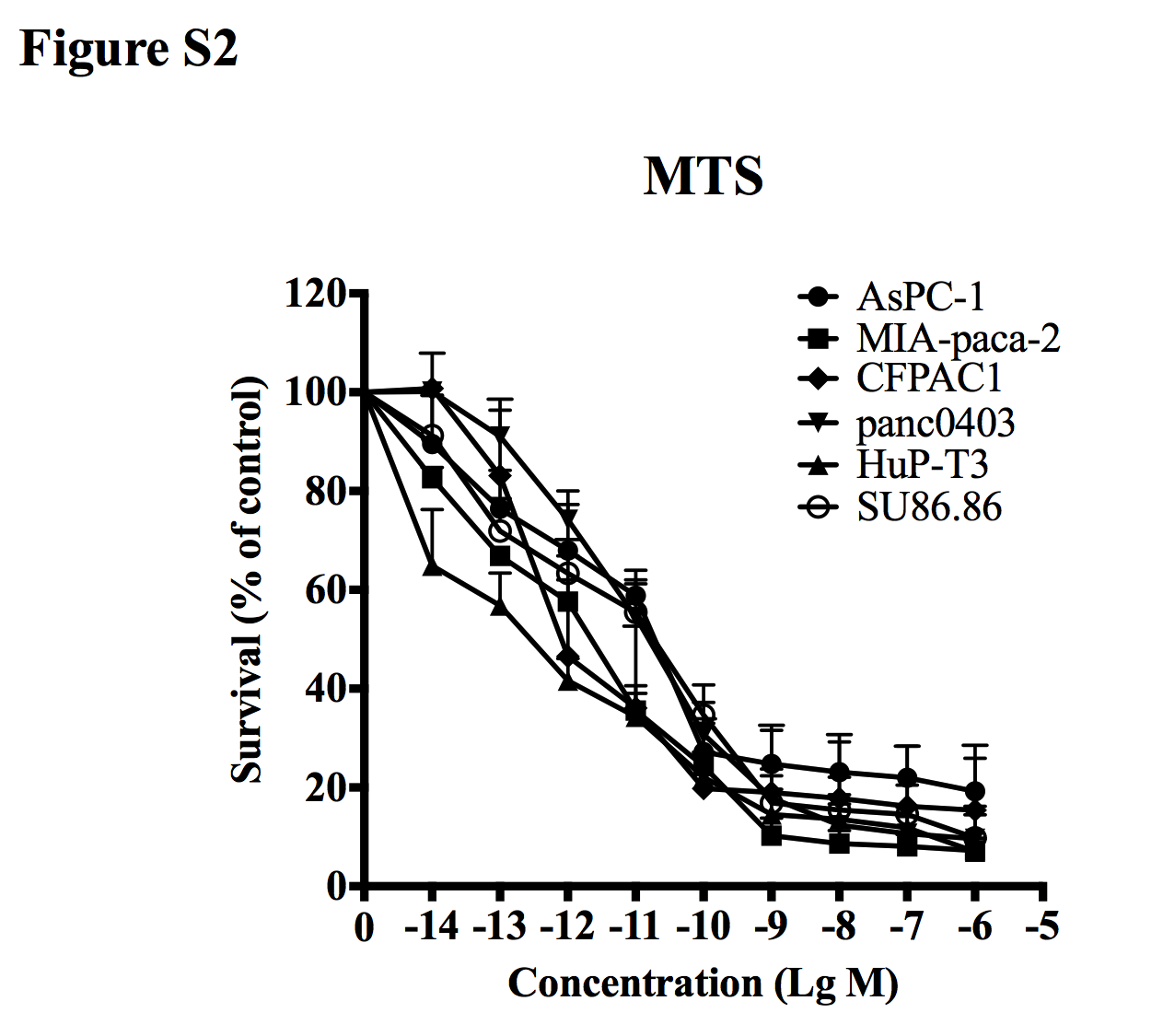

Supplement: Supplementary file 2 [file CAM4-8-643-s002.tiff]

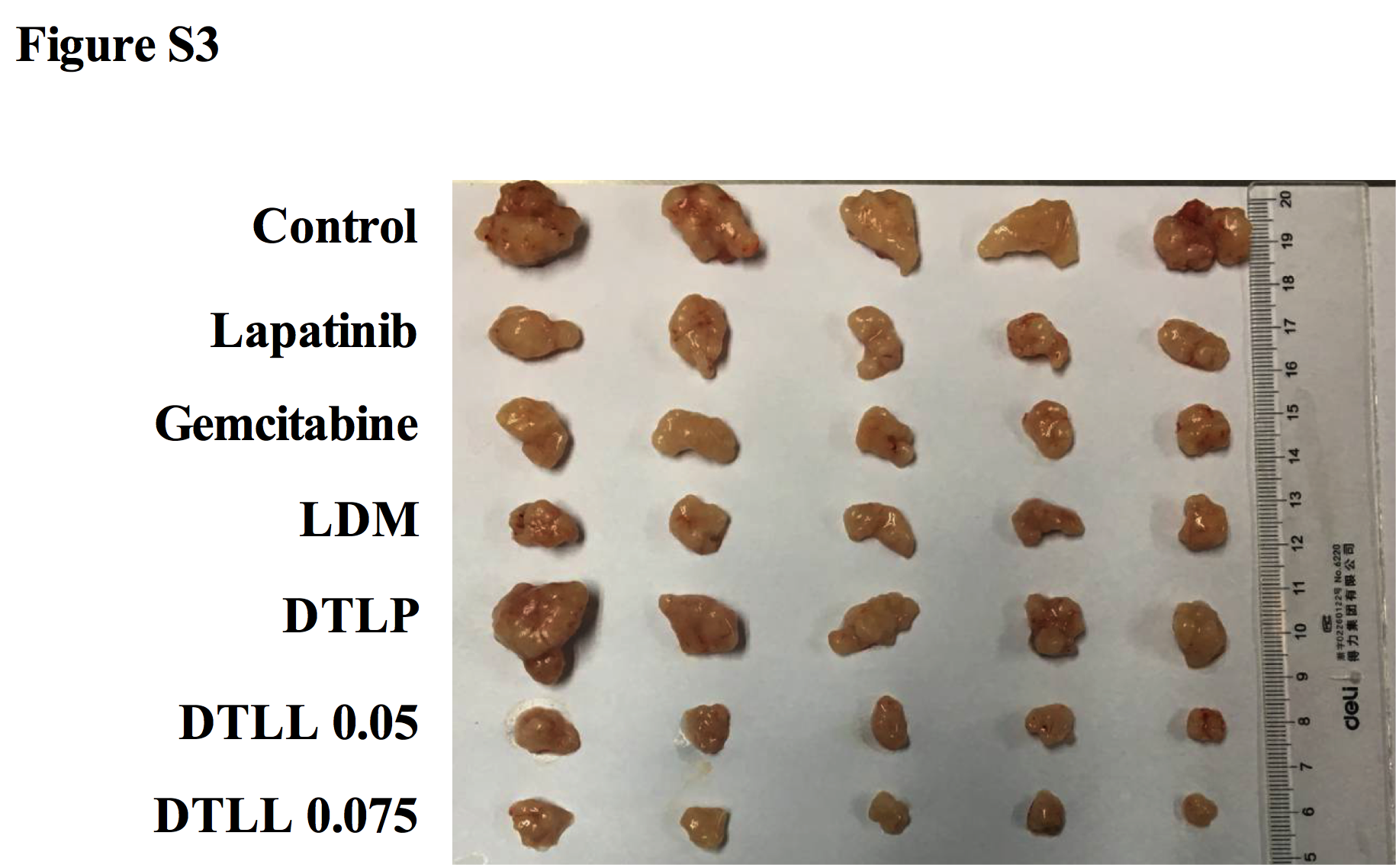

Supplement: Supplementary file 3 [file CAM4-8-643-s003.tiff]

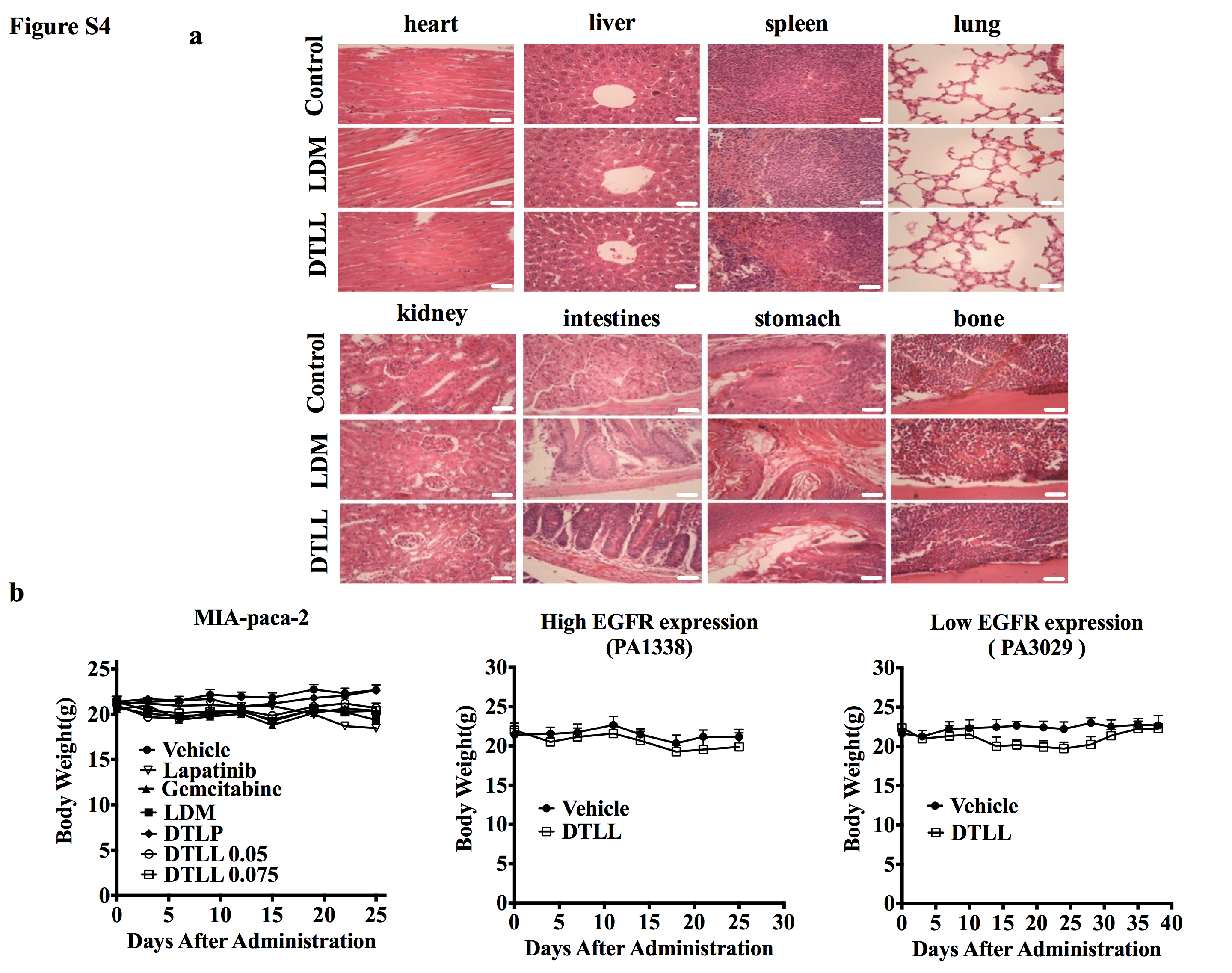

Supplement: Supplementary file 4 [file CAM4-8-643-s004.tiff]

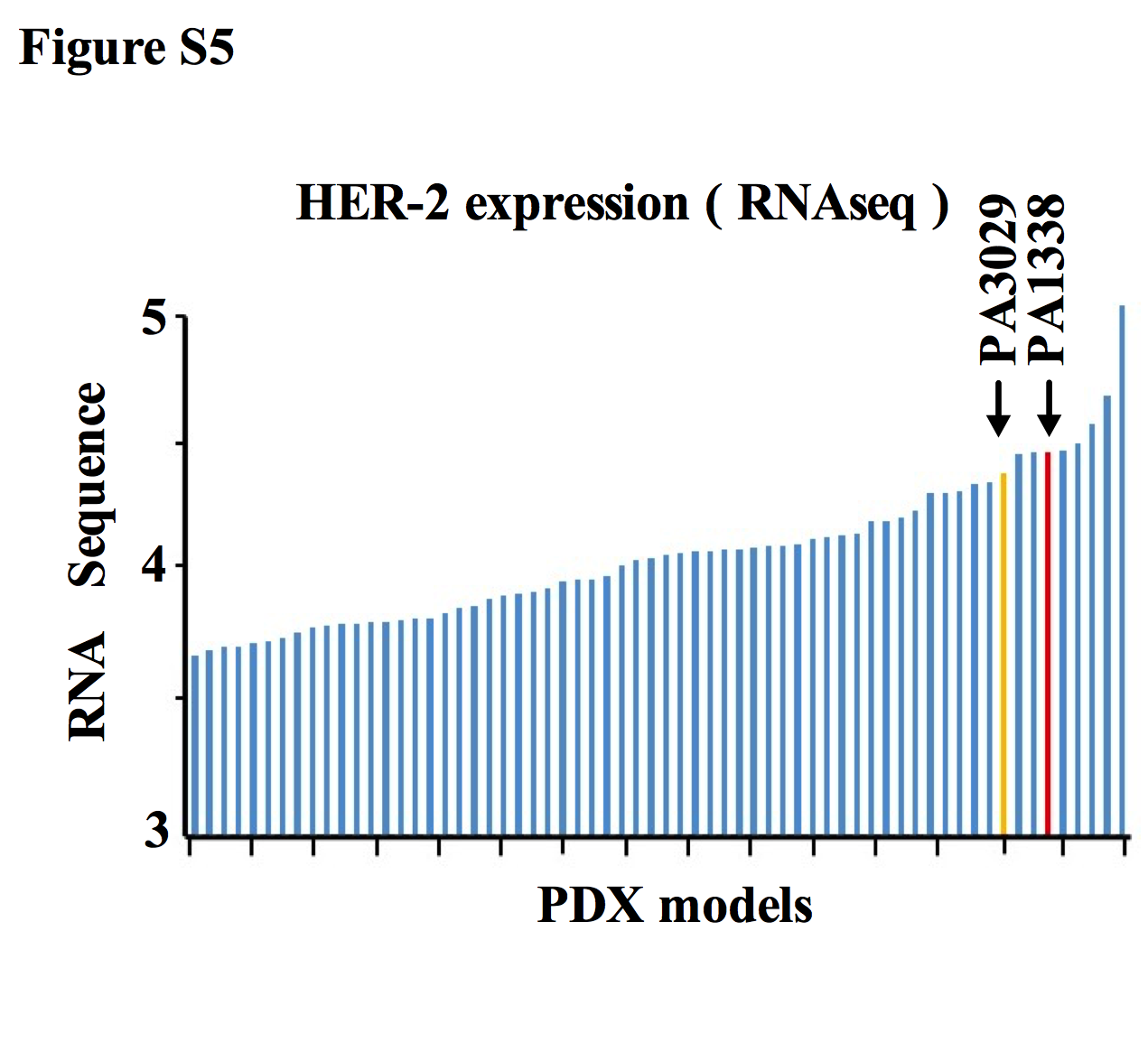

Supplement: Supplementary file 5 [file CAM4-8-643-s005.tiff]
